# Accurate HIV viral load measurement in primary health care settings using the cobas^®^ plasma separation card

**DOI:** 10.1371/journal.pone.0232122

**Published:** 2020-05-06

**Authors:** Adolfo Vubil, Ana Flora Zicai, Nádia Sitoe, Carina Nhachigule, Bindiya Meggi, Osvaldo Loquiha, Sofia Viegas, Nédio Mabunda, Lesley Scott, Ilesh Jani

**Affiliations:** 1 Instituto Nacional de Saúde, Marracuene, Mozambique; 2 Clinton Health Access Initiative, Maputo, Mozambique; 3 Department of Molecular Medicine and Haematology, School of Pathology, Faulty of Health Science, University of the Witwatersrand, Johannesburg, South Africa; Katholieke Universiteit Leuven Rega Institute for Medical Research, BELGIUM

## Abstract

**Introduction:**

Plasma is considered the gold standard for HIV viral load (VL) testing, however its use is challenging due to the need for phlebotomy and centrifugation services, as well as cold chain for transporting to laboratories for testing. The use of Dried Blood Spot (DBS) specimen has allowed a rapid expansion of antiretroviral therapy (ART) monitoring in remote areas in many African countries, however, the VL in DBS may overestimate the copies of viral RNA result at the clinically relevant range of 1000 copies/ml, due to proviral DNA and intracellular RNA. The characteristics of the cobas^®^ Plasma Separation Card (PSC) specimen are similar to fresh plasma (gold standard), so a better performance of HIV VL is expetected in PSC specimen and can be an alternative to DBS. This study aims to evaluate the performance of cobas^®^ PSC for VL testing at primary health care facilities in Mozambique.

**Methodology:**

HIV-1 infected adults on ART were enrolled consecutively in two health facilities in Mozambique, between August 2018 and October 2018. Capillary and venous cobas^®^ PSC, DBS and fresh plasma specimens were collected from each patient. All specimens were tested for VL using CAP/CTM v2.0. Sensitivity and specificity of viral load using DBS, capillary and venous PSC specimens were estimated. Viral load obtained in fresh plasma specimen was used as reference and a threshold of 1000 copies/ml was considered for the analyses.

**Results:**

From the total 613 patients included for the study, 2444 specimens including DBS (613), plasma (613), venous cobas^®^ PSC (609) and capillary cobas^®^ PSC (609) were collected and 2407 results were obtained. Sensitivity and specificity of the VL using venous cobas^®^PSC specimen at 1000 copies/ml threshold were 99.8% and 98.1% respectively, whereas for capillary cobas^®^ PSC sensitivity was 99.6% and specificity was 97.2%. For DBS VL, sensitivity was 96.9% and specificity was 81.8%. Misclassification rate was more prominent in DBS specimens (5.9%), but lower in venous and capillary cobas^®^PSC with a rate of 0.3% and 0.7% respectively.

**Conclusion:**

The cobas^®^ PSC specimen has improved performance over DBS for more accurate VL testing aligned with plasma testing. The use of this specimen type can increase the rates of reliable VL results and this will improve the quality of VL monitoring of HIV-positive patients in low-income settings.

## Introduction

The measurement of levels of HIV viremia in blood is the cornerstone of laboratory monitoring of anti-retroviral therapy (ART). In resource-limited settings, where most HIV infections occur, a mere 50% of patients on ART are currently routinely monitored using viral load testing [1]. Improved access to this critical laboratory assay is urgently needed as global and national initiatives aim to provide medical care to increasing numbers of HIV-infected individuals, including those living in hard-to-reach geographical areas.

HIV viral load determination is best achieved in fresh plasma specimens, with viral RNA quantification performed in laboratories using sophisticated equipment. Plasma must be separated from the cellular elements of blood within 6 hours post venesection, and then stored at -20°C or -80°C until testing in order to prevent degradation of RNA [2]. In resource-limited settings, especially in primary health care centers where most HIV-infected patients seek medical care, the separation of plasma and its timely transport to a usually small number of distant laboratories is an overwhelming task. This is mainly due to weaknesses in health systems, including shortage of human resources, lack of logistical capacity and poor cold chain infrastructure.

Many countries have implemented dried blood spots (DBS) to simplify the logistics of transporting clinical specimens to laboratories for viral load testing [3–7]. Whole capillary or venous blood is spotted onto filter paper cards to prepare DBS. This method of whole blood collection has been very successfully implemented for diagnosing HIV-infection among infants younger than 18 months through the qualitative detection of HIV-1 proviral DNA [8–10]. The comparative advantages of DBS include their ease of transportation, lack of cold chain needs and low biosafety requirements. However, HIV viral loads performed on DBS specimens are poorly correlated to those determined in plasma since whole blood contains also cell-associated RNA molecules and proviral DNA [6, 11–13]. Hence, viral load results from DBS specimens at clinicaly relevant threshold of 1000 copies/ml are frequently inaccurate and can mislead clinical-decision making. Viral load results from DBS specimens are more acurate at the threshold of 5000 copies/ml[14]

The cobas^®^ plasma separation card (Roche Diagnostics GmbH, Mannheim, Germany) (PSC) is a novel specimen collection and transport device, that allows for simultaneous collection of whole blood and plasma separation without the use of additional equipment. These cards maintain all the comparative advantages of DBS, while providing plasma as a matrix for laboratory assays. A recent study under controlled conditions in South Africa showed that viral load results determined using PSC correlated well with those generated using plasma [15]. Our study aimed at evaluating the performance of the PSC for HIV-1 viral load testing in patients attending primary health care facilities under routine health system conditions in Maputo City, Mozambique.

## Materials and methods

### Study design and participants

This cross-sectional study consecutively enrolled 613 HIV-infected adults receiving antiretroviral therapy (ART) between August 2018 and October 2018. The study was conducted at two primary health care centers in Maputo City, the Polana Caniço and the Primeiro de Maio Health Centers. These health centers provide a range of HIV-related clinical services, including ART initiation and follow-up. Both clinics routinely collect DBS specimens for viral load monitoring and send these to the Instituto Nacional de Saúde laboratories located approximately one hour driving distance from the health facilities for testing.

For each enrolled study participant, a single viral load testing was conducted on plasma, DBS and cobas^®^ PSC prepared from venous blood; and cobas^®^ PSC prepared from capillary blood. Technicians performing laboratory testing for one method were blinded to results generated by other methods. Standardised data collection forms were used to collect demographic data from all participants, including sex, age, ART regimen and length of time on ART.

### Specimen collection

The cobas^®^ PSC (*Roche Molecular Systems*, *Pleasanton*, *CA*, *USA*) is a blood collection and plasma stabilizing device that facilitates the filtration of whole blood into dried plasma spots. The device comprises a porous membrane which allows only plasma to filter through, while retaining all other blood components. After drying the specimen on the card, it can be transported under a wide range of environmental conditions to a central testing laboratory. Detailed description of how PSC works is available in a recent publication of Carmona et al (2019).

For this study, the cobas^®^ PSC (*Roche Molecular Systems*, *Pleasanton*, *CA*, *USA*) was prepared using both capillary and venous blood from each study patient. The capillary PSC specimen was collected using a single use safety lancet blue blade with a penetration depth of 2.0 mm for finger puncture. After puncturing, three capillary tubes marked 140μl provided with the test kit was used to collect a total of 420 μl of whole blood per card from each patient. After collection, blood in each capillary tube was transferred onto each of the three delineated areas of the card. In addition, 6.0ml venous blood was collected in a BD Vacutainer^™^K_2_EDTA (Becton, Dickinson and Company, 1 Becton Drive, Franklin Lakes, NJ 07417–1880, US) tube from each patient. Pasteur pipette was used to transfere one to two drops of whole blood onto each of delineated areas to get five full spots of the DBS card (Ahlstrom Germany GmbH) and an additional cobas^®^ PSC was prepared using venous blood. The remnant anticoagulated whole blood was transported to Instituto Nacional de Saúde laboratories within 6 hours post venesection, for plasma separation and storage at -80°C until viral load testing. All three cards (2 cobas^®^ PSC and 1 DBS) were placed overnight in a single-usage drying rack at room temperature. After drying, the cards were packed in separate gas impermeable zip-lock bags with desiccant and shipped to Instituto Nacional de Saúde laboratories for testing. All cards were stored at -80°C upon arrival at the testing laboratory. All staff involved in specimen collection for the study routinely collects DBS specimens for viral load, however in the context of this study were trained on PSC and DBS specimens collection and packing. Testing of study specimens (plasma and cobas^®^ PSC) followed the routine schedule of the laboratory, with DBS specimens having the priority as part of the medical management of patients.

### Specimen preparation

Whole blood anticoagulated specimens were centrifuged at 800-1600g for 20 minutes at room temperature to obtain plasma, of which 1100μl was used for viral load testing. One spot of DBS was eluted in phosphate buffered saline, pH 7.4 (1X) and incubated at room temperature for 30 minutes. After the incubation, specimens were manually homogenized and immediately loaded into the CAP/CTM v2 for testing using the free virus elution Roche protocol. For the cobas^®^ PSC, one spot was eluted in Sample Pre-Extraction (SPEX) solution (*Roche Molecular Systems*, *Pleasanton*, *CA*, *USA*), incubated in a thermomixer at 56°C and 1000 rpm for 10 minutes and then immediately loaded into CAP/CTM96 for testing.

### Viral load testing

Viral load testing on DBS specimens was performed as part of the routine patient monitoring, whereas plasma and PSC specimens were tested for study purposes. Testing for all specimens was carried out at the Instituto Nacional de Saúde using the Roche CAP/CTM 96 HIV-1 Quantitative Test v2 (Roche Molecular Diagnostics, Branchburg NJ, USA), according to the manufacturer’s instructions. The test definition files used for HIV viral load measurement was HI2PSC96 for cobas^®^ PSC specimens, IFS96CDC for DBS specimens and HI2CAP96 for plasma specimens. Interpretation of viral load results was performed according to manufacturers’ instructions, which establish 20, 400 and 738 copies/ml of viral RNA as the low limit of detection for plasma, DBS and PSC specimens, respectively. These differences on limit of detection are prominent, but not clinically relevant on perspective that all specimens with VL bellow 1000 copies/ml will have the same approach. However, a lower limit of detection of VL in PSC specimens can be used to monitor VL in patients with low-level viremia.

The reference laboratory routinely participates in and passed external quality assessment programs for viral load (provided by the US Centres for Disease Control and Prevention, Atlanta, USA) prior to and during the study period.

### Statistical analysis

When viral load results (copies/ml) were log10-transformed, those with non-quantifiable viral load results with values below the limit of detection (LOD) for each specimen type, were assigned a value of 1 copies/ml to enable quantitative log10 transformation and for graphic visualization. Values from plasma testing were regarded as the reference for calculating sensitivity, specificity and misclassification [2] of results obtained from DBS and cobas^®^ PSC specimens. A threshold of 1000 (3 log) copies/ml were considered for these calculations. All samples with viremia below 1000 copies/ml, regardless of the specimen-type used for testing, were considered as being virally suppressed as per WHO guidelines[16, 17]. Concordance correlation[18] and Bland-Altman[19] analyses were performed to determine precision and agreement between plasma, DBS, and venous and capillary cobas^®^ PSC. Specimen results that generated a reportable result across all specimen types were included in concordance correlation and these results >1 000copies/ml were included in the Bland-Altman analysis. Scatter plots were presented for visual representation of log transformed and untransformed data across specimen types. Analyses were performed using STATA 14.2 (StataCorp, Texas, USA) and MedCalc Statistical Software version 16.4.3 (MedCalc Software bvba, Ostend, Belgium).

### Ethical considerations

Ethical approval for the study was obtained from Mozambique’s National Health Bioethics Committee, with the reference number 297/CNBS, in July 2018. Written informed consent was obtained from each participant prior to conducting any study procedure. All patients were given a copy of the signed consent form, which contained information about the study as well as contacts details of the principal investigator and the ethics committee.

## Results

### Study population

A total of 613 consecutive patients attending the two health care facilities for routine ART monitoring between August 2018 and October 2018 were enrolled in the study. The median age of study participants was 41 years, and 65.0% were female (Table 1). Most patients (93.6%, 574/613) were receiving first line ART and the median time on ART was 51 months. The plasma viral load among participants showed that 50.9% (312/613) had non-quantifiable virus, 2.9% (18/613) had a viremia between 1000 and 10,000 copies/ml, and 15.5% (95/613) had a viremia >10,000 copies/ml.

**Table 1:** Patient demographic information (n = 613) across the two primary health care facilities.

		n	%
Sex	Male	205	33%
	Female	396	65%
	Not available	12	2%
ART regimen	Line 1	574	94%
	Line 2	26	4%
	Not available	13	2%
Age (years)	15–24	14	2%
	25–34	136	22%
	35–44	230	38%
	45–54	134	22%
	≥55	86	14%
	Not available	13	2%
**Median (IQR)**		**41**	**(35–49)**
Time on ART (months)	0–6	36	6%
	7–12	39	6%
	13–24	81	13%
	25–48	120	20%
	49–72	81	13%
	>72	204	33%
	Not available	52	9%
**Median (IQR)**		**51**	**(23–95)**
Plasma VL (cp/mL)	Not detected	312	51%
	<20	104	17%
	20–1000	80	13%
	1000–10 000	18	3%
	10 000–100 000	69	11%
	>100 000	26	4%
	Missing	4	1%
**Median (IQR)**		**54**	**(20–18972)**
Reason for VL	Routine	513	84%
	Virologic failure	99	16%
	Not available	1	0%

cp/ml—copies per mililiter

**Fig 1:**
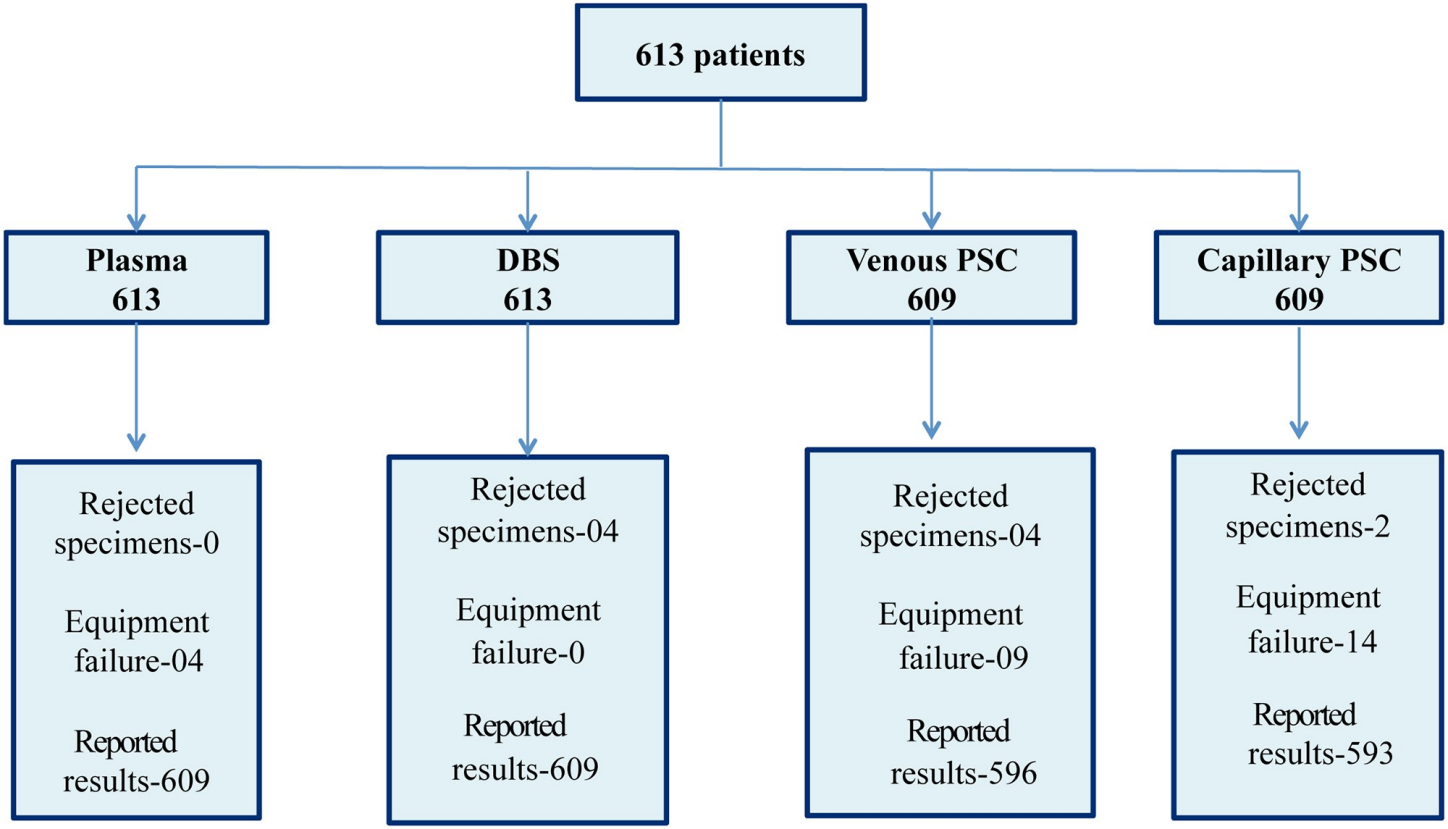
Overview of specimens collected and reportable results generated. The diagram illustrates the total number of patients recruited for the study, number of samples collected for each specimen type, number of samples rejected in the laboratory due to poor quality, number of results not reported due to equipment failure and the total number of results reported for each specimen type.

From the 613 patients included in the study, 2444 specimens including DBS, plasma, venous PSC and capillary PSC were collected, and 2407 (98.5%) generated reportable results (Fig 1). Overall, 0.41% (10/2444) specimens were rejected at the reference laboratory due to poor quality and 1.1% (27/2434) failed to report a result due to equipment failure. From the rejected specimens, four were DBS, four venous PSC and two capillary PSC. For all specimens, the reason of rejection was spot not completely full, which means that the quantity of blood transferred onto the delineated area of the card was not sufficient.

### Specimens testing

At the time of the study, the standard-of-care for HIV viral load testing used for patient management in Mozambique was DBS, which took preference upon specimen arrival in the testing laboratory. The median number of days between specimen collection and testing for DBS was 48 days (range: 1–91), for plasma was 57 days (range: 17–129), for venous cobas^®^ PSC was 66 days (range 13–130) and for capillary cobas^®^ PSC was 58 days (range 13–130). No bias in batch testing for any particular viral load category was evident from internal review of measuring the difference between DBS, cobas^®^ PSC and plasma over the time of storage at -80°C and testing. The analysis of viral load difference plotted against number of days to testing, to ensure -80°C storage did not affect the viral load yielded a random scatter, showing no trend in specimen type and result difference with the reference plasma.

In addition, the laboratory work flow was not dependent on specimen type for loading specimens into the equipment. The CAP/CTM instrument software used in the laboratory was adaptable to plasma, DBS or cobas^®^ PSC testing protocols, and various specimen types were tested in the same run on the same day.

### Performance of the cobas^®^ plasma separation card

**Fig 2:**
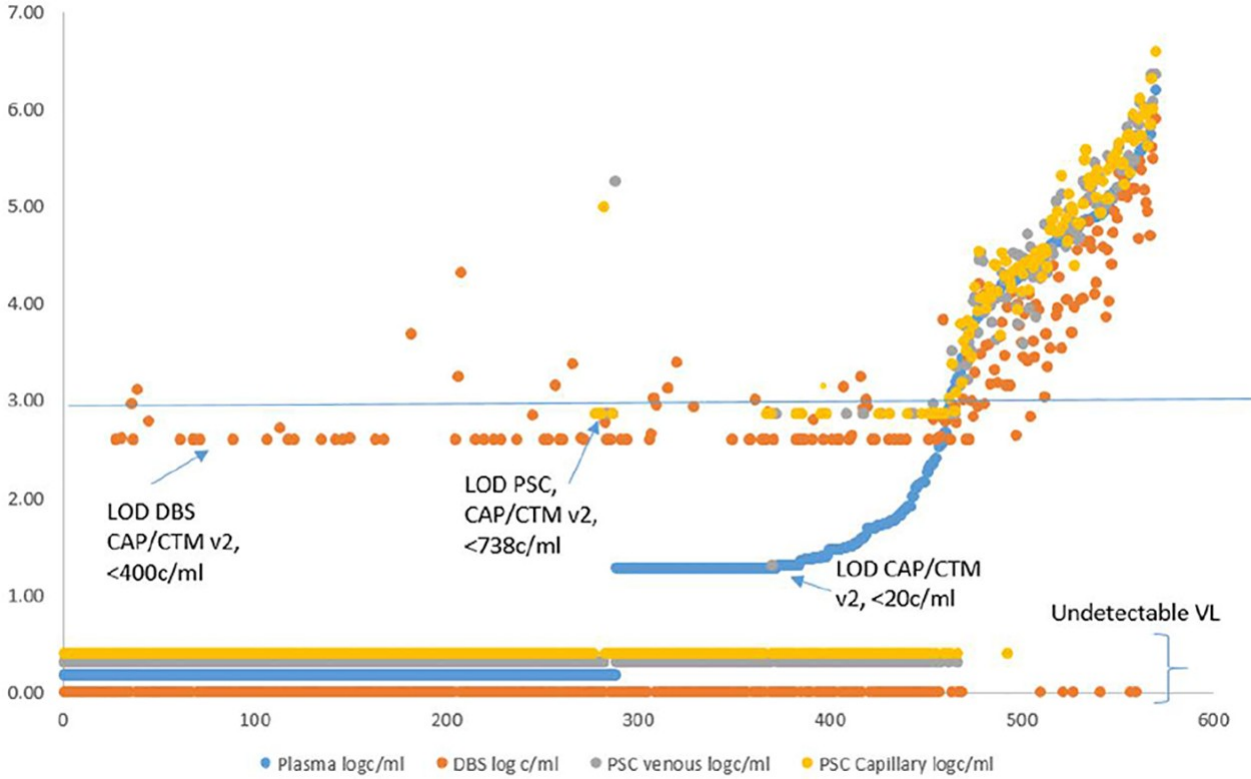
HIV VL results for plasma, DBS, capillary and venous PSC specimens. A scatter plot illustrating quantitative HIV viral load results generated across testing (n = 570) with the cobas^®^ PSC (capillary and venous collection) and DBS compared to plasma viral load testing. The vertical axis is increasing viral load (log copies/ml) and the horizontal axis is specimen number sorted by the reference method (increasing plasma viral load). The legend lists the 4 specimen types. Sorted specimen numbers 1–288 yield an undetectable VL from plasma specimen, followed by specimen numbers 289–372 yielding a VL <20 copies/ml, and specimen number 373–570 yield VL results>20 copies/ml.

Fig 2 illustrates the absolute HIV VL results from study participants and shows that DBS specimens with VL >1000 copies/ml underestimate the true HIV viral load as measured by the reference plasma specimen. In addition, the DBS reports several HIV VL results >1000 copies/ml when the plasma VL reference values are undetectable. The cobas^®^ PSC prepared either from capillary or venous blood specimens, generate the same trend of higher values than plasma VL in specimens >1000 copies/ml.

**Fig 3:**
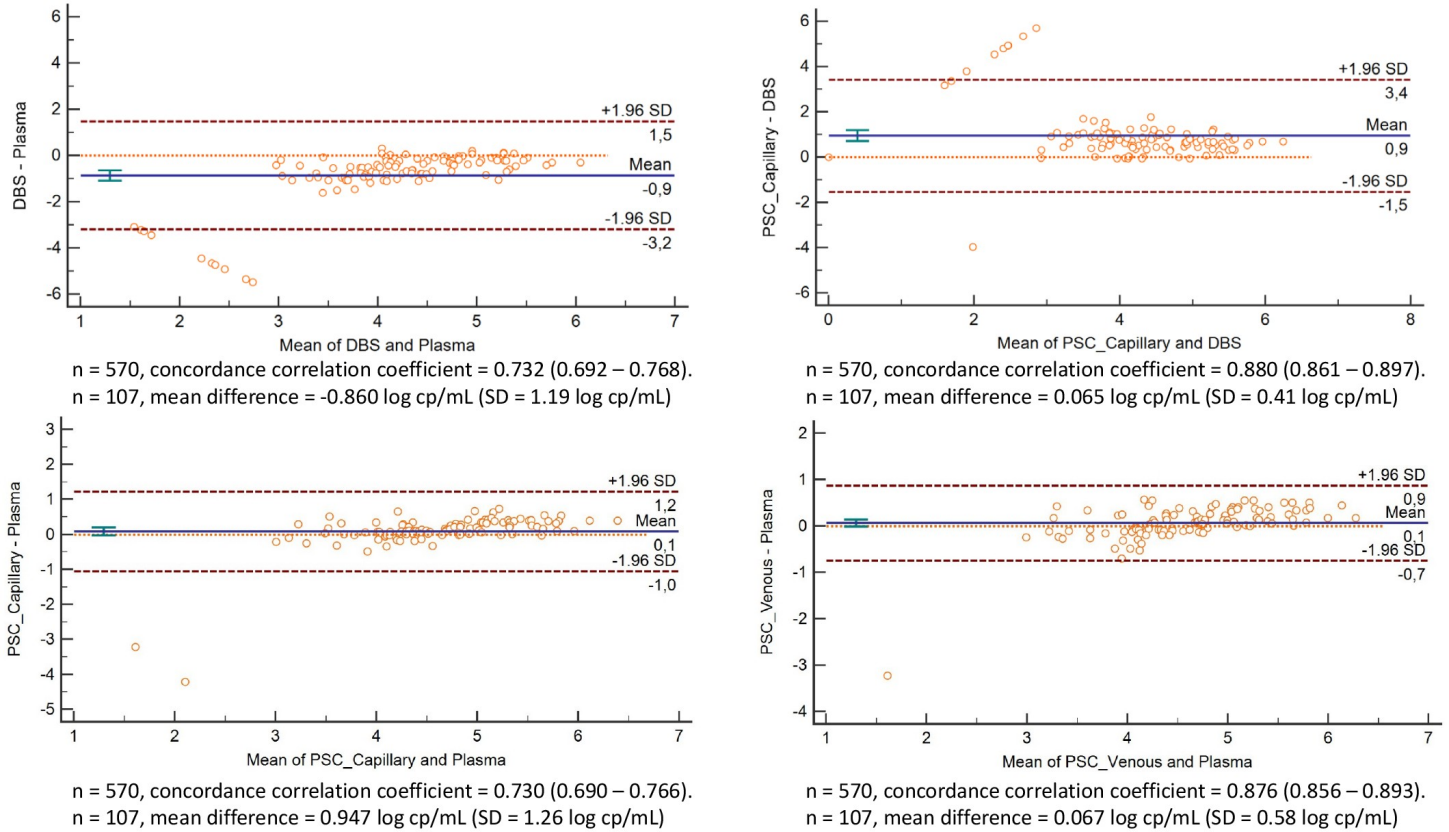
Scatter plots of absolute difference in log copies/ml VL versus mean VL (bland-altman analysis). The vertical axis is log difference between test and reference. The bias (Blue line), LOA (Limits Of Agreement in dotted red lines), SD (Standard Deviation in aqua error bar) are indicated on the plots. The legend also includes specimen number and concordance correlation coefficients.

The clinical relevance was further investigated by measuring the sensitivity, specificity and misclassification rate as outlined in Table 2 and the absolute bias by Bland-Altman and concordance correlation in Fig 3.

**Table 2:** Sensitivity, specificity and misclassification rate (95% confidence interval) for capillary cobas^®^ PSC, venous cobas^®^ PSC and dried blood spot (DBS) specimens relative to 1000 c/ml plasma viral load.

	DBS	cobas^®^ PSCc	cobas^®^PSCv
Sensitivity	97.0% (95.1% 98.3%)	99.8% (99.8% 100%)	100% (99.2% 100%)
Specificity	81.4% (73.0% 88.1%)	97.3% (92.4% 99.4%)	98.2% (93.6% 99.8%)
PPV	95.8% (93.7% 97.4%)	99.4% (98.2% 99.9%)	99.6% (98.5% 100%)
NPV	86.0% (77.9% 91.9%)	99.1% (95.0% 100%)	100% (96.7% 100%)
Misclassification rate	5.9% (4.2% 8.1%)	0.7% (0.2% 1.7%)	0.3% (0.0% 1.2%)
False positive rate	18.6% (11.9% 27.0%)	2.7% (0.6% 7.6%)	1.8% (0.2% 6.4%)
False negative rate	3.0% (1.7% 4.9%)	0.2% (0.0% 1.2%)	0.0% (0.0% 0.8%)

DBS—dried blood spots; cobas^®^ PSCc—plasma separation card prepared using capillary blood; cobas^®^PSCv—plasma separation card prepared using venous blood; PPV—positive predictive value; NPV—negative predictive value

The sensitivity of the cobas^®^ PSC (99.8%, capillary and 100% venous) in identifying virological failure and success at the clinically relevant threshold of 1000 copies/ml, as determined by plasma, was statistically significantly better than DBS (97%), as shown by non-overlapping confidence intervals. This is similarly evident with the specificity, with DBS (81.4%) performing poorly compared to the cobas^®^ PSC (97.3%, capillary and 98.2% venous). This performance is further compounded by the higher misclassification rate for DBS (5.9%), compared to lower rates of misclassification for the cobas^®^ PSC (0.7%, capillary and 0.3% venous).

Among the quantifiable VL results, the concordance correlation coefficient showed poorer accuracy and precision for measuring VL using DBS (0.732) than cobas^®^ PSC (0.876 capillary and 0.880 venous). The bias among quantifiable results across all specimen types again outlines the reduced accuracy of the DBS compared to the reference plasma. DBS overall generates 0.86 log copies/ml lower results than plasma VL, with several outliers (n = 9) in the clinically relevant range at 1000 copies/ml across the 107 paired specimens, and high variability (standard deviation of the bias >1.0log copies/ml) in the bias. The cobas^®^ PSC (either venous or capillary collection) displayed minimal bias (<0.09 copies/ml) compared to plasma with only 3 outliers across 107 data pairs. Fig 3 further illustrates the potential bias between DBS and cobas^®^ PSC capillary, that would be anticipated within a testing program where cobas^®^ PSC were to replace DBS in ART monitoring of routine patients. Approximately 1.0 log copies/ml difference in VL results would be reported across the quantifiable range of VL values for patient care, with the cobas^®^ PSC overall generating higher VL values than DBS and therefore aligning the true VL result from a cobas^®^ PSC more closely with plasma VL.

Based on the data presented in Fig 2, and the good agreement between cobas^®^ PSC and plasma, it is evident that the cobas^®^ PSC is a matrix suitable for use for VL testing. Based on the sensitivity, misclassification rate and absolute bias analysis, we identified two discordant results for venous PSC, three for capillary PSC and 36 for DBS.

## Discussion

The laboratory monitoring of ART in many sub-Saharan settings is currently performed on DBS, as this type of specimen overcomes many of the logistical challenges faced by resource-limited health systems. Our study shows that DBS constitutes a sub-standard alternative to plasma viral load, and demonstrates that accurate determination of viral load is feasible when cobas^®^ PSC is used to obtain patient specimens under field conditions.

The relatively low bias (<0.09 copies/ml) displayed for venous and capillary cobas^®^ PSC at the plasma 1000 copies/ml range is an acceptable bias, and in line with the manufacturer’s definition of acceptability of <0.3log copies/ml [20, 21]. The bias generated by cobas^®^ PSC is similar to the viral load differences reported previously comparing split plasma specimens across different testing platforms or assay versions [22, 23], and similar to other plasma separation devices applied to HIV VL testing[24]. On the contrary, the bias seen on DBS viral load (>1.0log copies/ml) in our study is notably higher than the values considered to be acceptable, and similar to other findings of the performance of DBS for HIV VL testing [24].

The better performance of the cobas^®^ PSC is further evidenced by the very low misclassification rates of viral load values below 1000 copies/ml, with rates of 0.7% and 0.3% for capillary and venous cobas^®^ PSC specimens, respectively, as opposed to 5.9% for DBS specimens. This is most likely because the use of the cobas^®^ PSC eliminated the interference of cell-associated viral nucleic acid and the over-quantification of HIV genetic material, a factor that impacts the measurement of viral load around the critical threshold of 1000 copies/ml [11–13].

The low limit of detection of 738 copies/ml in the cobas^®^ PSC specimens is considerably higher than the 400 copies/ml and 20 copies/ml obtained with the DBS and fresh plasma, respectively. These limits are defined for the CAP/CTM system used in our study, but the differences are likely to be seen in other testing systems. Nevertheless, the limit of detection for the cobas^®^ PSC is still below the threshold of 1000 copies/ml used to currently define viral suppression as per WHO recommendations [16, 17]. Moreover, the limit of detection defined for DBS specimens does not take into account the loss of specificity caused by cell-associated nucleic acid in whole blood.

In most countries of sub-Saharan Africa, specimens for HIV viral load testing are collected at health facilities and sent to a relatively small number of reference laboratories for testing [25]. For example, according to the DISA Lab Viral Load National Database, in Mozambique ten Laboratories were tasked with testing the 801,155 specimens collected in 2018. Consequently, most laboratories have a relatively long turnaround time for viral load testing. In this context, it is critical that specimens be stable for periods of at least two months. In this study, the cobas^®^ PSC was tested between 13 and 130 days post venesection without a loss in RNA stability, but with storage prior to testing at -80°C.

In this study we did not investigate the stability of PSC in a variety of temperature ranges, but previous stability data has shown that the cobas^®^ PSC can be stored for 56 days in a temperature range between 2 to 30°C without significant variance on the HIV viral load result [15].

Nurses in two primary healthcare centres performed the collection of the cobas^®^ PSC specimens for this study, attesting to the feasibility of implementing this new method in resource-limited health systems. The procedures for collecting cobas^®^ PSC specimens are similar to those for DBS, which health professionals throughout sub-Saharan Africa have been using in HIV viral load testing and early infant diagnosis for many years [6, 8–10]. Additionally, in this study viral load testing using cobas^®^ PSC specimens was executed in a laboratory that routinely tests for viral load and early infant diagnosis, with no additional equipment required.

## Conclusion

In conclusion, the implementation of the cobas^®^ PSC in primary healthcare centers is feasible, when prepared using either capillary or venous blood. HIV viral loads determined using this new technology for specimen collection and transport are accurate, and therefore, cobas^®^ PSC constitutes a good alternative to fresh plasma for the laboratory monitoring of patients undergoing ART in resource-limited settings.
